# Accelerated Phase Chronic Myeloid Leukemia and Treatment Free Remission Maintained After Five Years of Nilotinib: A Case Report

**DOI:** 10.3389/fonc.2021.696253

**Published:** 2021-06-11

**Authors:** Isabella Capodanno, Elisabetta Lugli, Katia Codeluppi, Mariapina Faruolo, Enrica Bellesia, Riccardo Valli, Francesco Merli

**Affiliations:** ^1^ Azienda Unità Sanitaria Locale-IRCCS di Reggio Emilia, Struttura Complessa di Ematologia, Reggio Emilia, Italy; ^2^ Azienda Unità Sanitaria Locale-IRCCS di Reggio Emilia, Laboratorio Chimico Clinico e di Endocrinologia, Reggio Emilia, Italy; ^3^ Azienda Unità Sanitaria Locale-IRCCS di Reggio Emilia, Servizio di Anatomia Patologica, Reggio Emilia, Italy

**Keywords:** nilotinib, chronic myeloid leukemia, tyrosine kinase inhibitor, accelerated phase, treatment free remission

## Abstract

The present article reports the case of a patient presenting with chronic myeloid leukemia, diagnosed during the accelerated phase (>20% blasts in peripheral blood samples and megakaryocyte agglomerates in the bone marrow). The subject was treated with first-line therapy with the tyrosine kinase inhibitor nilotinib and reached complete clinical and molecular remission (according to the European Leukemia Net-ELN-criteria), which persisted over five years of treatment. Five years after discontinuation of nilotinib (ten years from diagnosis), the patient is in good clinical condition, with no traces of BCL-ABL1 at molecular evaluation (molecular response, MR^5^). The case is discussed in the setting of current literature, providing an overview on chronic myeloid leukemia and a discussion on treatment options available.

## Introduction

Chronic myeloid leukemia (CML) is a clonal stem cell disorder caused by the BCR-ABL translocation, affecting individuals generally over the age of 60 years and accounting for approximately 15% of all leukemias in Europe. In recent years, therapeutic advances of the tyrosine kinase inhibitor (TKI) family have allowed a good control of CML and, in several cases, complete remission (both clinical and molecular); however if left untreated or undiagnosed, CML progresses from its chronic phase into its accelerated phase, eventually leading to the highly fatal blast phase (1–3).

In patients achieving a deep molecular response with a median follow-up of 77 months after discontinuation of imatinib, the molecular recurrence-free survival was 43% at 6 months and 38% at 60 months (4). Moreover, the possibility to discontinue treatment has been extensively investigated in retrospective and prospective studies that have shown that a consistent proportion of patients, ranging from 30 to 70%, who discontinue treatment after having achieved a deep molecular response may remain treatment-free for an as-yet undefined period of time (2, 5–15).

To date, however, there is still a lack of comparative data on the efficacy of different treatment policies and different TKIs for TFR, leaving open the debate on the choice of treatments. Some data will likely come three years from now by the ongoing GIMEMA CML trial (SUSTRENIM), which is investigating the 5-year TFR rate in newly diagnosed CML patients treated first-line with nilotinib or with imatinib followed by switch to nilotinib in the case of non-optimal response (16).

Also, several retrospective and prospective studies have extensively investigated the possibility of discontinuing treatment in patients who have achieved a deep molecular response, reporting treatment-free survival rates between 30 and 70% (2, 5–15). In particular, treatment with imatinib yielded a molecular recurrence-free survival of 43% at 6 months and 38% at 60 months, with a median follow-up of 77 months after discontinuation (4).

However, because data available on treatment-free remission (TFR) are only for patients in the chronic phase and not for CML in advanced phases, current clinical guidelines and recommendations suggest TFR for chronic-phase patients only (17–19).

In an attempt to contribute to filling this information gap, here we report a case of a patient treated with the TKI nilotinib, from June 2011 to January 2016, diagnosed in accelerated phase CML, who showed complete clinical and molecular remission persisting also five years after treatment discontinuation.

## Case Description

**Figure 1:**
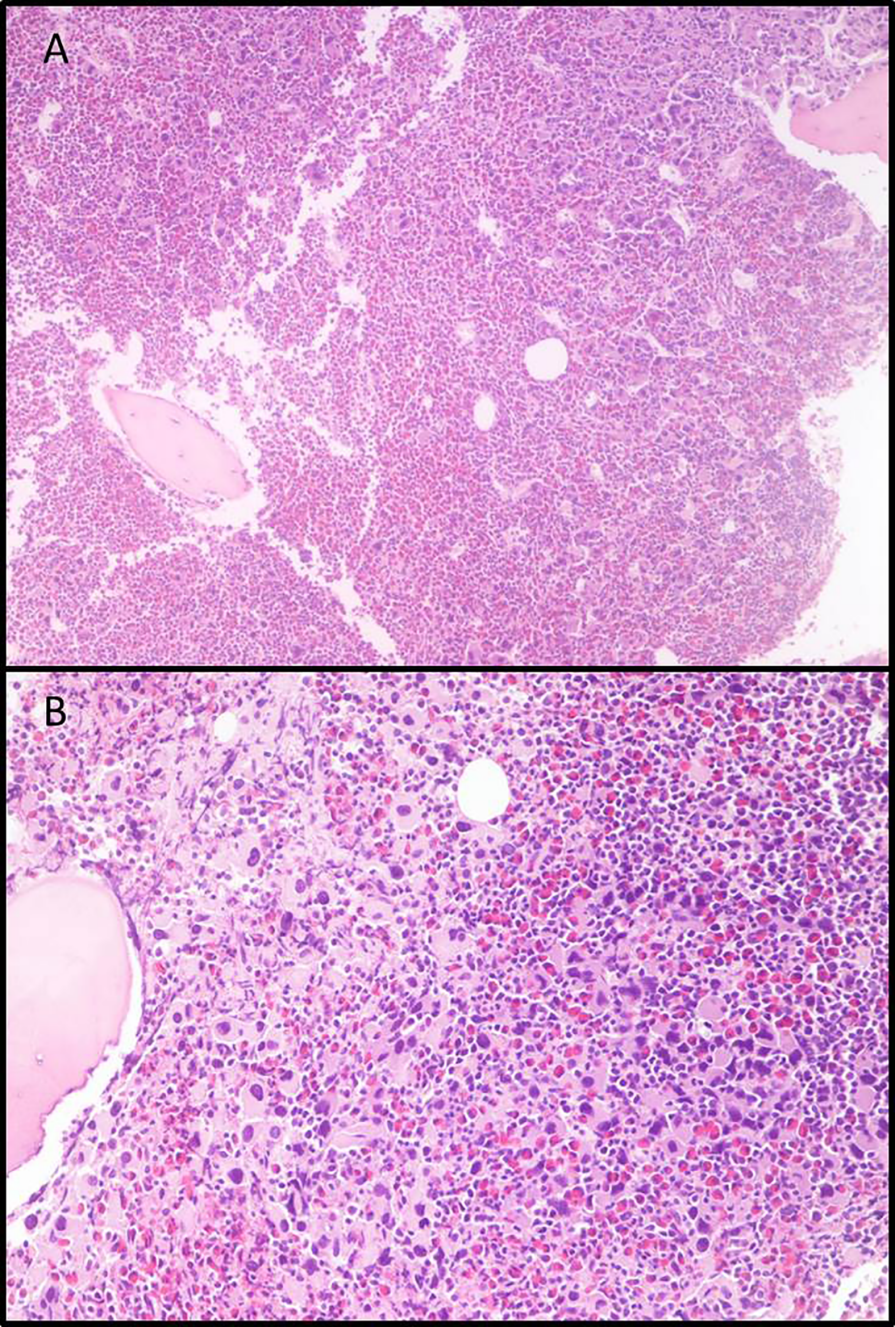
Bone marrow biopsies. **(A)** Eosinophilia and micro-megakaryocyte proliferation. Evident signs of fibrosis, original magnification 5×; **(B)** Micro-megakaryocytes characterized by hypo-lobated nuclei, original magnification 20×.

In June 2011, a 54-year-old woman was admitted to our Center, with leukocytosis, grade I anemia (Hb 11.4 g/dl), increased platelet count (3.903.000/µl), LDH 527 U/l, and Raynaud episodes mainly in her feet. Laboratory tests confirmed the increase in white blood cell count, up to 22.540 mcg/l, with neutrophils 36%, lymphocytes 14%, metamyelocytes 3%, eosinophils 7%, basophils 33%, blasts 7%, myelocytes 2%, and erythroblasts 2%. All other laboratory tests were in range. The molecular analysis of JAK2 did not reveal any mutation, whereas BCR-ABL1 was positive for the transcript “b2a2”. Bone marrow biopsy revealed large megakaryocyte conglomerates with abnormal elements (**Figure 1**); elevated blasts count (>20%) in repeated peripheral blood samples was also found, leading to the diagnosis of CML in accelerated phase according to ELN classification. Cytogenetic evaluation showed the standard BCL-ABL1 translocation 9/22, without any additional alteration. Sokal risk score (151.73) and Hasford risk score (2589.19) were elevated.

Treatment with second-generation TKI nilotinib started in June 2011 at the dose of 400 mg twice daily. It was effective and well tolerated: after one month of treatment, in July 2011, the patient showed complete hematologic response according to the current guidelines. After 3 months of treatment, in November 2011, a complete cytogenetic response (CCyR), as well as a deep molecular response (DMR) was obtained, with BCR-ABL1 transcript level detectable in peripheral blood sample = 0.0003% IS. This response was confirmed and further improved during the following months, and in particular, BCR-ABL1 transcript was undetectable after 12 months of treatment (MR^5^). From January 2015 to January 2016, the patient maintained the deep molecular response. In January 2016, due to a personal urgent request from the patient, therapy was discontinued although our advice was to continue the treatment according to current guidelines. For the following 6–12 months, the BCL-ABL1 transcript could not be dosed (MR^5^), whereas minimal oscillations in its levels were detected afterwards, but always maintaining a stable DMR (MR^4.5^/MR^5^) up to January 2017; afterwards the transcript level remained 0% (MR^5^) until the last control in February 2021.

Regarding safety, our patient showed a very good tolerance to treatment with nilotinib: she reported only mild increase in lachrymation, with eye-discomfort after prolonged laptop use. Thus, we were able to maintain treatment for the recommended period of five years. As by standard recommendations, the patient is still under evaluation within a schedule of periodic follow-up appointments.

## Discussion

TKI therapy is the current standard of care for patients with CML with second-generation compounds such as nilotinib and dasatinib showing deeper and faster response rates compared to imatinib in the first-line setting. Compared to imatinib, nilotinib is more potent, featuring a 30- to 50-fold higher affinity for the BCR-ABL1/ATP binding site (20). In particular, nilotinib has shown to be more effective than imatinib in achieving molecular response, as well as cytological response, at 12 months, with lower incidence of progression to the accelerated phase and/or the blastic one (21).

The overall response to CML treatment is evaluated based on several reference criteria parameters (including clinical/hematological, cytogenetic, and molecular responses) to be reached and maintained afterwards in order to define an optimal, warning or failure status, as outlined in current guidelines at different timepoints (18, 22, 23). Specifically, the ELN, European Leukemia Net defined criteria for each response, as well as suggesting the most appropriate treatment according to the risk factors. According to these GLs (18), a deep molecular response (MR^4^ or MR^5^) is considered as achieved with low level of BCR-ABL1 gene transcript (<0.01 and <0.0032% respectively) upon standardized real-time quantitative polymerase chain reaction. Although patients achieving a complete molecular remission cannot be considered healthy, therapy may be discontinued in a certain percentage of CML patients. TKI discontinuation in patients with durable DMR is considered to be safe. In addition to the STIM trial (2), many other studies reported TFR success rates ranging from 30 to 70% in selected CML patients, after first or second generation TKIs (2, 5–15). In the ENEST freedom trial, investigating TFR after a median of 3.6 years of nilotinib treatment in first line CML, demonstrated that 51.6% of patients at 48 weeks remained in MMR or better after discontinuation (5). A recent update of this study shows the durability of the obtained result, also after 192 weeks after discontinuation (24). Factors supporting treatment discontinuation mainly depend on disease/patient characteristics, such as the presence of BCR-ABL1 typical transcripts at diagnosis, a treatment duration of at least 5 years, the achievement of a MR4.5 and its durability for at least 2 years (22, 23).

Guidelines also suggest proper follow-up includes frequent molecular monitoring, with monthly visits for the first six months and then every three months thereafter. In general, though, the reference criteria in TKI discontinuation eligibility outside of a clinical trial are quite strict as shown by both the ESMO and the very recently updated NCCN guidelines (17). A shared recommendation is to exclude patients with high Sokal risk or with a previous history of accelerated phase or blast phase CML. Noteworthy to mention is that while the inclusion of high-risk chronic phase CML patients had been permitted in TFR trials, a diagnosis (or previous diagnosis) of accelerated phase CML represented an exclusion criteria in the same studies perhaps due to tolerability issues also in this subpopulation. Yet, despite discontinuation being a clinical practice, to date there are no data available reporting the outcome of accelerated phase CML patients undergoing TFR.

A recent study (25) including 75 patients with accelerated phase CML at diagnosis, twenty-three of whom were treated with nilotinib upfront, has suggested the association between early response at 3 and 6 months to be a strong determinant of long-term outcome. This is also compatible with observations from our experience with a patient with accelerated phase CML and elevated risk scores: after a 5-year treatment with nilotinib, the patient reached a successful TFR, maintaining deep molecular remission for more than five years (total observation period 10 years).

The achievement of an operational cure as a treatment goal has changed dramatically the whole disease paradigm in CML, increasing focus on quality of life and avoiding long-term organ toxicities. With new generation TKIs and novel TFR trials and strategies, these results are improving and are spreading year after year into clinical practice. However, a not secondary challenge would be not only to improve TFR rate, but also to extend the possibility to be eligible to a safe TFR for a wider population, with the aim of limiting toxicity and reducing costs of treatment.

## Data Availability Statement

The original contributions presented in the study are included in the article/supplementary material. Further inquiries can be directed to the corresponding author.

## Ethics Statement

Written informed consent was obtained from the individual(s) for the publication of any potentially identifiable images or data included in this article.

## Author Contributions

IC and EL offered the case. IC prepared the manuscript. FM revised the manuscript. KC and MF edited the manuscript. RV prepared **Figure 1**. EB did the molecular analysis. MW revised the English language. All authors contributed to the article and approved the submitted version.

## Conflict of Interest

The authors declare that the research was conducted in the absence of any commercial or financial relationships that could be construed as a potential conflict of interest.
